# From Basic Biology to Patient Mutational Spectra of *GATA2* Haploinsufficiencies: What Are the Mechanisms, Hurdles, and Prospects of Genome Editing for Treatment

**DOI:** 10.3389/fgeed.2020.602182

**Published:** 2020-11-26

**Authors:** Cansu Koyunlar, Emma de Pater

**Affiliations:** Department of Hematology, Erasmus MC, Rotterdam, Netherlands

**Keywords:** *GATA2*, Inherited bone marrow failure syndrome, MDS, AML, *GATA2* haploinsufficiency syndrome, genome editing, HSCs, autologous HSC transplantation

## Abstract

Inherited bone marrow failure syndromes (IBMFS) are monogenetic disorders that result in a reduction of mature blood cell formation and predisposition to leukemia. In children with myeloid leukemia the gene most often mutated is *Gata binding protein 2* (*GATA2*) and 80% of patients with *GATA2* mutations develop myeloid malignancy before the age of forty. Although *GATA2* is established as one of the key regulators of embryonic and adult hematopoiesis, the mechanisms behind the leukemia predisposition in *GATA2* haploinsufficiencies is ambiguous. The only curative treatment option currently available is allogeneic hematopoietic stem cell transplantation (allo-SCT). However, allo-SCT can only be applied at a relatively late stage of the disease as its applicability is compromised by treatment related morbidity and mortality (TRM). Alternatively, autologous hematopoietic stem cell transplantation (auto-SCT), which is associated with significantly less TRM, might become a treatment option if repaired hematopoietic stem cells would be available. Here we discuss the recent literature on leukemia predisposition syndromes caused by *GATA2* mutations, current knowledge on the function of *GATA2* in the hematopoietic system and advantages and pitfalls of potential treatment options provided by genome editing.

## Introduction

IBMFS are a heterogeneous cluster of disorders manifested by an ineffective blood production and concurrent cytopenias that eventually result in a hypoplastic bone marrow (BM). These syndromes constitute an increased propensity to develop hematological malignancies such as myelodysplastic syndrome (MDS) and acute myeloid leukemia (AML) (Dokal and Vulliamy, 2010; Wilson et al., 2014; Cook, 2018). Mutations in *GATA2* are the most common genetic defects in pediatric MDS (Spinner et al., 2014). *GATA2* is one of the master regulators of blood production and patients that carry a mutation in one of the two alleles of *GATA2* often manifest with immunodeficiency syndromes and increased lifetime risk for MDS/AML (Wlodarski et al., 2016; Donadieu et al., 2018; McReynolds et al., 2018). Once malignant transformation becomes overt, survival rates are below 50% (Spinner et al., 2014). Due to the inherited mutation, allo-SCT is the only curative treatment option for these patients (Simonis et al., 2018; van Lier et al., 2020). Unfortunately, the use of allo-SCT is compromised by TRM and not applicable for patients who have not progressed to leukemia yet. Uncovering the *modus operandi* of *GATA2* and other (epi)genetic factors in the complex network of blood regulation is essential to design non-invasive and preventive treatment options for IBMFS patients.

Genome editing strategies, especially the implementation of clustered regularly interspaced short palindromic repeat/associated protein 9 (CRISPR/Cas9) nuclease platforms, improve rapidly and progress toward efficient therapies for several genetic diseases (Cong et al., 2013; Mali et al., 2013; Anzalone et al., 2019). In this review, we will summarize clinical symptoms of *GATA2* haploinsufficiency patients and results from *Gata2* experimental models to inspect the function of *GATA2* in leukemogenesis. Our aim is to explore the potential and pitfalls of genome editing methods to treat *GATA2* deficiency syndromes in the light of current technologies.

## The Transcription Factor *GATA2*

**Figure 1:**
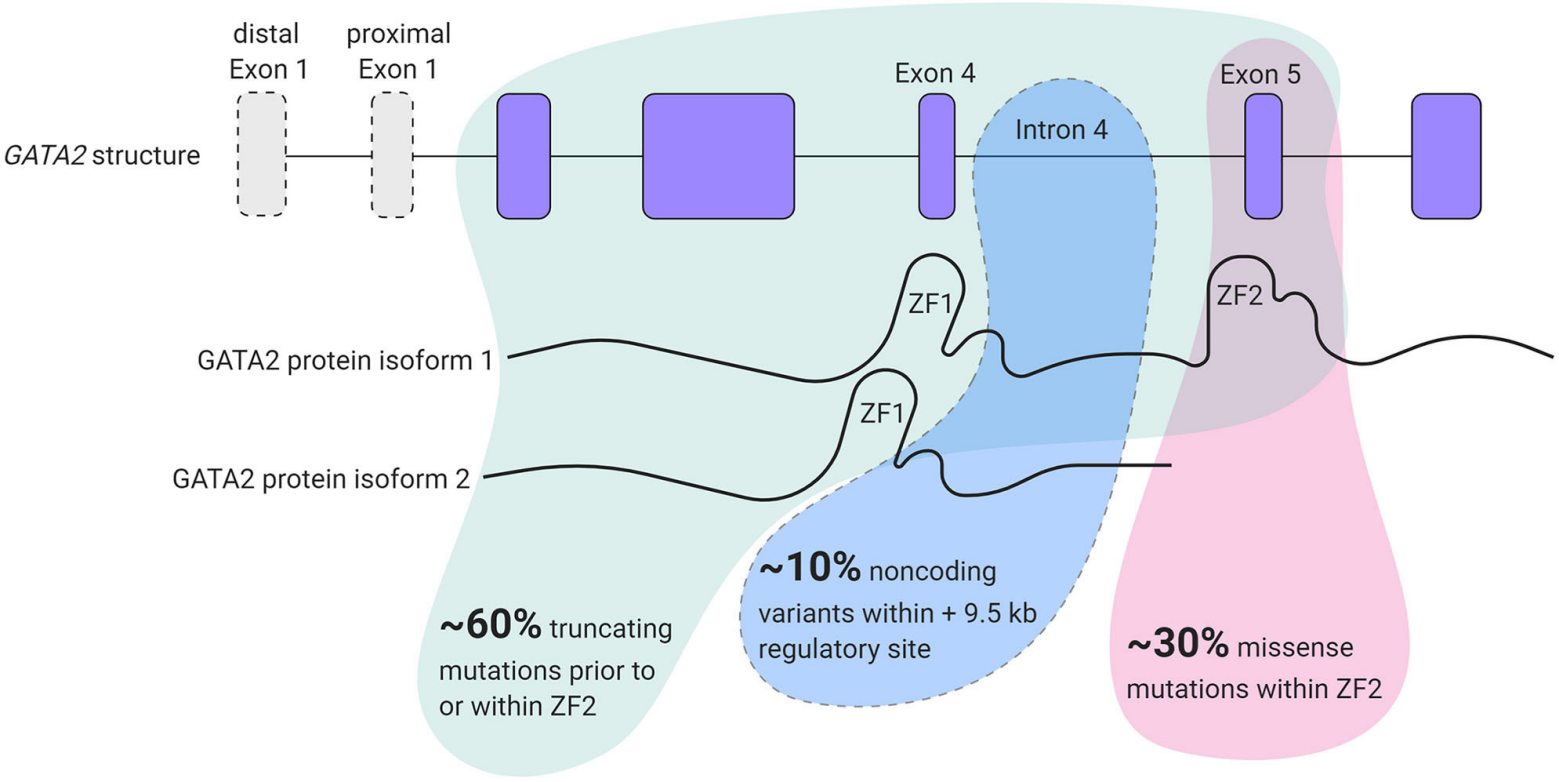
*GATA2* locus organization and overview of mutation types found in *GATA2* haploinsufficiency patients.

*GATA2* is a zinc finger transcription factor that contains 2 first exons; a hematopoietic and neuronal cell specific distal first exon and a proximal first exon that is utilized ubiquitously. These two transcript variants encode the same protein (Minegishi et al., 1998; Pan et al., 2000). *GATA2* binds a highly conserved (A/T)GATA(A/G) DNA sequence and other protein partners through two multifunctional zinc finger (ZF) domains; ZF1 and ZF2 that are encoded by exon 4 and exon 5, respectively (Evans and Felsenfeld, 1989; Alfayez et al., 2019). Two *GATA2* protein isoforms can be formed, one lacking exon 5 and consequently lacking the ZF2 domain (Vicente et al., 2012) (Figure 1). To date, the functional consequence of this remains unclear.

## Germline *GATA2* Mutations

In 2011, four different studies described germline heterozygous *GATA2* mutations in a total of 44 patients with various syndromes; monocytopenia and mycobacterial infection (MonoMAC) syndrome (Hsu et al., 2011), monocyte, B cell, NK cell and dendritic cell deficiencies (DCML) (Dickinson et al., 2011), Emberger Syndrome, which is characterized by primary lymphedema with a predisposition to AML (Ostergaard et al., 2011) and familial MDS/AML predisposition (Hahn et al., 2011). Distinct clinical perspectives discerned in these studies coalesce under the theme of the loss of one allele of *GATA2* resulting in the *GATA2* haploinsufficiency syndrome, which can present with immunodeficiency, lymphedema and 80% predisposition to develop MDS/AML.

Taken together, 60% of patients present with a truncating mutation in *GATA2* before the ZF2 domain and 30% of patients present with a non-synonymous mutation in ZF2. However, some patients develop MonoMAC syndrome without mutations in the coding region of *GATA2* but have reduced *GATA2* expression levels (Hsu et al., 2013). These patients harbor mutations in the intronic region, specifically in intron 4. Mutations in this region abrogate the function of a conserved +9.5 cis-element, that regulates *GATA2* transcription levels resulting in *GATA2* haploinsufficiency (Hsu et al., 2013) and intron 4 mutations represent 10% of all *GATA2* haploinsufficiency cases (Wlodarski et al., 2017) (Figure 1). *GATA2* mutations are also present in a subset of patients with chronic neutropenia and aplastic anemia (AA) (Townsley et al., 2012; Pasquet et al., 2013). However, BM of AA patients with *GATA2* mutations encompasses noticeably different types of altered hematopoietic populations than idiopathic AA patients, such as the complete loss of lymphoid progenitors and atypical megakaryocytes (Ganapathi et al., 2015).

Both familial and sporadic mutations in the coding and cis-regulatory regions of *GATA2* are found and are the underlying cause in 15% of advanced and 7% of all pediatric MDS cases (Wlodarski et al., 2016). Most of these mutations can be found in the ClinVar database (https://www.ncbi.nlm.nih.gov/clinvar). Currently, despite the improving definition of the phenotypic characteristics of *GATA2* deficiency syndromes and high penetrance of myeloid malignancy, the mutational background and phenotypic outcome observed in these patients do not correlate, suggesting that additional events are important for disease progression (Collin et al., 2015; Wlodarski et al., 2016). Evidence for this is found in a cohort of pediatric MDS-*GATA2* patients that acquired additional somatic mutations in *ASXL1, RUNX1, SETBP1, IKZF1*, and *CRLF*2 genes, which resulted in an increased progression to AML. Furthermore, 72% of adolescents with MDS and monosomy 7 had an underlying *GATA2* mutation (Wlodarski et al., 2016; Fisher et al., 2017; Yoshida et al., 2020).

## Somatic *GATA2* Mutations

Although truncating germline *GATA2* mutations occur most often, a few somatic mutations are reported that phenocopy germline loss-of-function mutations (Sekhar et al., 2018; Alfayez et al., 2019). These cause a relatively milder form of the immunodeficiency phenotype observed in germline mutant *GATA2* patients, along with a common presentation of AML, atypical chronic myeloid leukemia and in some cases acute erythroid leukemia (Ping et al., 2017; Sekhar et al., 2018; Alfayez et al., 2019).

Somatic *GATA2* mutations are found both in ZF1 and ZF2 and all patients with somatic *GATA2* mutations harbor mutations in other genes, predominantly *CEBPA* with an incidence of 18–21% (Fasan et al., 2013; Hou et al., 2015; Theis et al., 2016). In one cohort of AML patients, ZF1 but not ZF2 mutations in *GATA2* closely associate with biallelic *CEBPA* mutations (Tien et al., 2018). This implies that ZF1 is crucial for *GATA2* function in disease progression in combination with *CEBPA* mutations.

## The Function of the Transcription Factor *GATA2* in Mammalian Hematopoiesis

### The Function of *GATA2* in Embryonic Hematopoiesis

In mouse, homozygous deletion of *Gata2* results in 67% lethality at embryonic day (E) 10.5 and none survive beyond E11.5, due to severe anemia. Chimeras of WT and *Gata2*^−/−^ embryonic stem (ES) cells show that *Gata2*-null cells cannot contribute to hematopoiesis in adult blood, fetal liver, BM and thymus revealing a requirement for *Gata2* in embryonic hematopoiesis (Tsai et al., 1994). Besides the embryonic lethality of *Gata2*-null embryos, the number and function of hematopoietic stem and progenitor cells from germline heterozygous *Gata2* mutant mice at E10.5–E12 is impaired (Ling et al., 2004).

Both in human and mouse embryos, *Gata2* is expressed in a specialized endothelial cell population called hemogenic endothelium (HE) and in the first transplantable hematopoietic stem cells (HSCs) that differentiate from HE (Marshall et al., 1999; Yokomizo and Dzierzak, 2010; Eich et al., 2018; Vink et al., 2020). Conditional deletion of *Gata2* in HE cells resulted in reduced hematopoietic cluster formation in the embryo and long-term repopulating HSCs were not formed. Conditional deletion of *Gata2* in HSCs induced apoptosis indicating that *GATA2* is required both for HSC generation and maintenance (de Pater et al., 2013).

*Gata2* expression is regulated by the enhancer activity of multiple conserved cis-regulatory elements. The disruption of the +9.5 element of *Gata2* impaired vascular integrity and formation of HSCs from HE in the mouse embryo (Lim et al., 2012; Gao et al., 2013).

Although both number and functionality of HSCs were reduced in embryonic *Gata2* haploinsufficiency, it is yet to be discovered whether and how the propensity for MDS/AML observed in *GATA2* haploinsufficiency patients is influenced by these early embryonic functions.

### The Function of *GATA2* in Adult Bone Marrow Hematopoiesis

The function of *GATA2* in adult hematopoiesis is still abstruse. In BM, *Gata2* is highly expressed in HSCs and downregulated during lineage commitment (Akashi et al., 2000; Miyamoto et al., 2002; Guo et al., 2013). HSCs in the BM of *Gata2*^+/−^ mice are impaired in number and functionality as shown by serial transplantation assays (Rodrigues et al., 2005; Guo et al., 2013). In addition, *Gata2*-heterozygosity in BM HSCs is associated with a decreased proliferation ability together with increased quiescence and apoptosis (Ling et al., 2004; Rodrigues et al., 2005). Moreover, *Gata2* haploinsufficiency reduces the function of granulocyte-macrophage progenitors but not of other myeloid committed progenitors (Rodrigues et al., 2008). However, *Gata2*^+/−^ mice do not develop MDS/AML. This makes it difficult to study the contribution of *GATA2* haploinsufficiency to leukemic progression in these models.

On the other hand, *Gata2* overexpression results in the self-renewal of myeloid progenitors and blocks lymphoid differentiation in mouse BM (Nandakumar et al., 2015). In addition, overexpression of *GATA2* in human ES cells (hESC) promotes proliferation in hESCs, but quiescence in hESC-derived HSCs (Zhou et al., 2019). Furthermore, increased *GATA2* expression is also observed in adult and pediatric AML patients with poor prognosis (Ayala et al., 2009; Luesink et al., 2012; Vicente et al., 2012; Menendez-Gonzalez et al., 2019). These findings indicate that, next to its tumor suppressor role, *GATA2* might act as an oncogene when overexpressed.

## Genome Editing: A Cure for *GATA2* Haploinsufficiencies?

### GATA2 Repair Strategies

**Figure 2:**
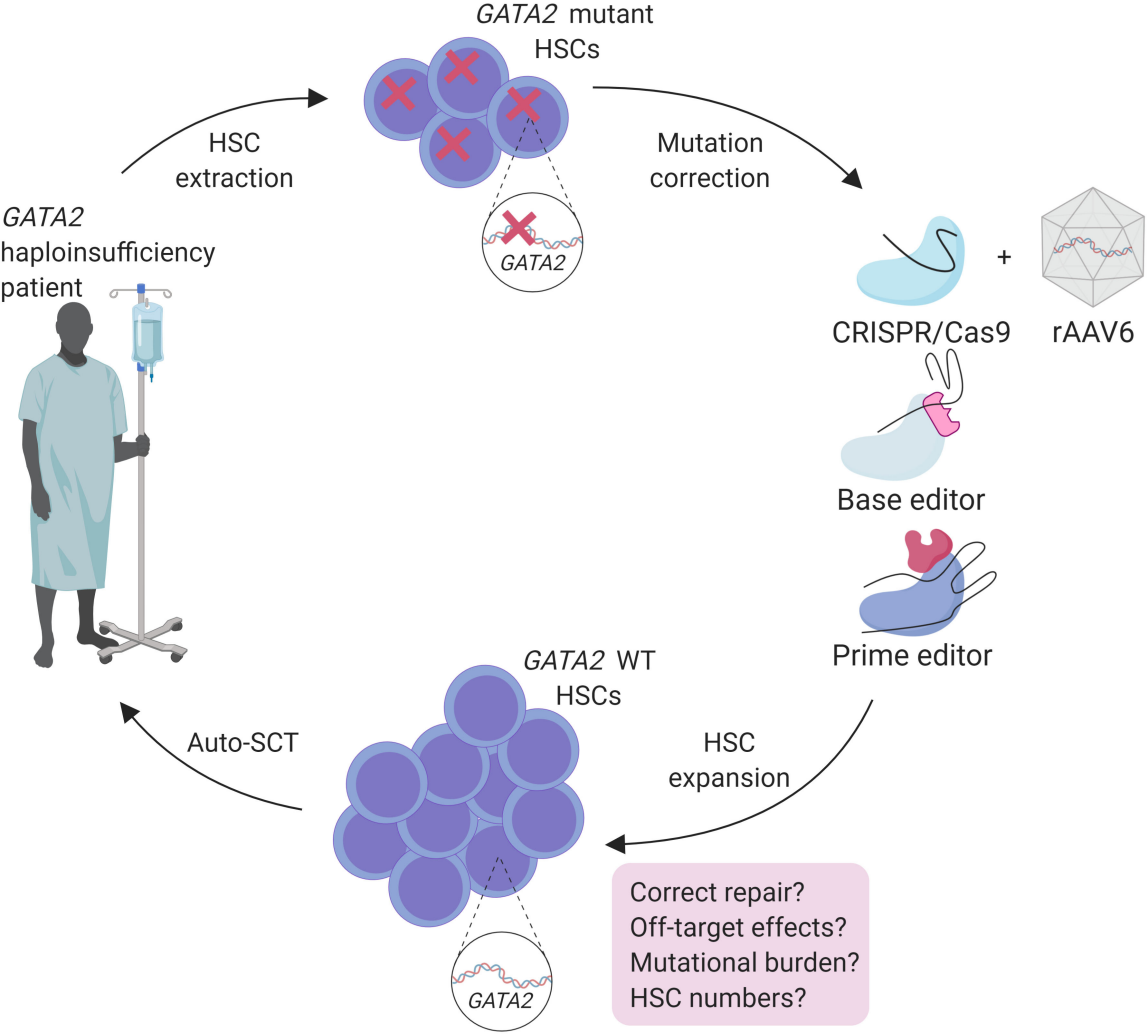
Treatment strategy for genome engineering of autologous HSC of *GATA2* haploinsufficiency patients with safety considerations.

Allo-SCT is a powerful approach to treat malignancies in *GATA2* haploinsufficiency patients (Simonis et al., 2018; van Lier et al., 2020). However, finding a matched donor and TRM compromises the use of allo-SCT and is therefore not suitable before the onset of malignancy (Bogaert et al., 2020). Regulation of *GATA2* expression is crucial in HSCs and in leukemia predisposition. This makes overexpression of WT *GATA2* using lenti-viral transgenic approaches not suitable as gene therapy method. An auto-SCT approach, after *ex vivo* correction of the underlying patient specific *GATA2* mutation by genome editing tools is possibly a more effective treatment option for these patients (Figure 2).

Genome editing, since it was pioneered in the previous century, is developing meteorically as a revolutionary therapeutic tool for genetic defects, including hematological disorders (Xie et al., 2014; Hoban et al., 2016; De Ravin et al., 2017; Orkin and Bauer, 2019) CRISPR/Cas9, a part of the bacterial acquired immune system, was adapted as a breakthrough genome engineering technology and has since been extensively used to engineer eukaryotic cells in basic research and holds great potential for gene therapy (Gasiunas et al., 2012; Jinek et al., 2012; Cong et al., 2013; Barrangou and Doudna, 2016). CRISPR/Cas9 mediated genome editing relies on sequence specific guide RNAs that assemble with Cas9 protein to create double strand breaks (DSBs) in the targeted sequence. DSBs activates cell intrinsic repair mechanisms if the cell is to undergo proliferation and repaired by one of two mechanisms: non-homologous end joining (NHEJ) in which random insertions/deletions (InDels) are introduced or homology-directed repair (HDR) which uses the other DNA strand as template to restore its original sequence. This system can be hijacked by providing an exogenous repair template containing any desired sequence. Because HDR is rare, a selection cassette can be inserted for positive selection of the desired repair (Doudna and Charpentier, 2014).

Because heterogeneity of mutations in *GATA2* haploinsufficieny patients (https://www.ncbi.nlm.nih.gov/clinvar), these mutations would need to be restored at the endogenous locus, requiring HDR as repair mechanism. Therefore, optimizing an editing strategy by using large HDR donor templates that cover various *GATA2* mutation regions found in patients or the whole gene, containing homologous regions covering several exons, could provide treatment for a substantial group of *GATA2* patients. An efficient method for gene correction in HSCs with CRISPR/Cas9 and large HDR donor delivered by rAAV6 (adeno-associated viral vectors of serotype 6) was used to correct a *HBB* gene mutation causing sickle cell disease and has potential to correct *GATA2* mutations in HSCs using the same strategy (Dever et al., 2016; DeWitt et al., 2016; Bak et al., 2018) (Figure 2).

### Hurdles

*GATA2* haploinsufficiencies result in a diminished number of HSCs in both embryonic and adult stages. Additionally, HDR mediated repair works with low efficiencies and studies showed that it is more efficient in hematopoietic progenitor cells rather than long-term repopulating HSCs (Genovese et al., 2014; Hoban et al., 2015). Together this implicates the biggest hurdle to treat *GATA2* haploinsufficiency patients would be to obtain sufficient number of corrected HSCs for auto-SCT. An enrichment method, possibly a reporter-based selection followed by an *ex vivo* expansion of *GATA2*-corrected HSCs, could potentially solve this problem. For this purpose, small molecule drugs promoting *ex vivo* expansion of HSCs, like SR1 or UM171, could be used to obtain higher number of corrected HSCs prior to auto-SCT (Boitano et al., 2010; Fares et al., 2014).

Furthermore, in *GATA2* haploinsufficiency patients, additional mutations in other genes could be the driver of leukemia which brings challenges to treat these patients by only correcting the mutant *GATA2* allele. Therefore, a preliminary genetic screening for additional mutations should be compulsory in *GATA2* haploinsufficiency patients to elucidate if correcting only the mutant *GATA2* allele would eliminate the disease phenotype of the patient.

Another hurdle when using genome editing tools for clinical applications is the off-target effects (OTEs) that might occur in undesired parts of the DNA. Detection of OTEs with whole genome sequencing are often challenging due to high background of random reads in combination with low sequence depth (<10-fold) (Kim et al., 2015). More screening strategies for OTEs, like GUIDE-seq (Genome wide, Unbiased Identification of DSB Enabled by sequencing), CIRCLE-seq (Circularization for *in vitro* Reporting of Cleavage Effects) and DISCOVER-seq (Discovery of *in situ* Cas Off-targets and VERification by Sequencing), are shown to overcome these obstacles and could be used to efficiently identify OTEs that might result from *GATA2*-editing strategy before its clinical translation (Tsai et al., 2015, 2017; Wienert et al., 2019).

### Prospects

Fortunately, recent improvements of CRISPR/Cas9 genome editing may overcome some of these hurdles for patient applications. Base editing methods are developed by the addition of enzymes to Cas9 to provide single base pair changes without making DSBs (Komor et al., 2016; Gaudelli et al., 2017). Although base editing can correct point mutations that are also found in *GATA2* patients, the off-target effects caused by the broad activity of cytidine deaminases used in this method should be considered carefully (Zuo et al., 2019; Yu et al., 2020). More recently Anzalone et al. (2019) described prime editing that introduces specific insertions, deletions and point mutations to a variety of genomic regions with high efficiency without DSBs. Prime editing was successfully used in human cells to correct mutations that cause sickle cell disease and Tay-Sachs disease and only 1–10% of prime-edited cells are found to have unwanted off-target InDels throughout the genome (Anzalone et al., 2019). These recent advances in genome editing techniques anticipate the improvement of a safer and more efficient correction of the patient mutations in HSCs prior to auto-SCT, and should be considered for the treatment of *GATA2* haploinsufficiencies (Figure 2).

Currently, the minimum level of donor chimerism necessary to reverse the disease phenotype in *GATA2* haploinsufficiency patients remains unclear (Hickstein, 2018). For sickle cell disease however, it was shown that clinical benefits might be observed when as few as 2–5 HSCs are engrafted (Walters et al., 2001; DeWitt et al., 2016). Interestingly, an asymptomatic germline *GATA2* mutant individual acquired a somatic mutation reversing the harmful *GATA2* mutation. This resulted in a selective advantage of the corrected HSCs and prevented from developing malignancy (Catto et al., 2020). Together this implicates having a few mutation-corrected HSCs might already have clinical significance for *GATA2* haploinsufficiency patients.

CRISPR/Cas9 technology has been approved in patient treatment for various types of malignancies including hematological diseases (https://clinicaltrials.gov). Currently, clinical trials are performed where CRISPR/Cas9 is used to remove erythroid expression of the fetal hemoglobin repressor *BCL11A* in the treatment of hemaglobinopathies, implicating a highly promising potential for genome editing to treat various hematological disorders (Orkin and Bauer, 2019; The Lancet Haematology, 2019).

Careful consideration of possible challenges discussed for *GATA2* haploinsufficiency patients could lead to a beneficial clinical translation of genome editing to treat these patients in the near future.

## Discussion

Although *GATA2* haploinsufficiency depletes the HSC compartment in humans and mice, the function of *GATA2* haploinsufficiency in MDS/AML progression is poorly understood. A possibility could be that *GATA2* haploinsufficiency provides a fertile ground for the emergence of additional mutations in HSCs and these acquired mutations promote leukemogenesis. Evidence that support this hypothesis is the inconsistent penetrance of leukemia in *GATA2* haploinsufficiency patients that cannot be explained solely by the mutations in the *GATA2* locus and MDS/AML patients with germline *GATA2* mutation presented with additional mutations which are linked to hematological malignancies (Wlodarski et al., 2016; Fisher et al., 2017; Yoshida et al., 2020). In order to understand the concept of fertile ground as a driver of MDS/AML in *GATA2* deficiency syndromes, more fundamental research is needed to reveal the clonal origin (embryonic and/or adult) of leukemogenic driver mutations to help us choose an appropriate time frame and strategy to treat these patients using genome editing. If leukemic driver mutations arise early during hematopoietic development, targeting leukemic clones will be challenging.

*in vivo Gata2*^+/−^ models have not developed an MDS/AML phenotype (Ling et al., 2004; Rodrigues et al., 2005). This could be due to differences governing HSC mechanisms in these models or due to differences in lifespan, infection status, genetic background or a combination of these factors. Perhaps aged *Gata2*^+/−^ models could provide more insight, since this would challenge the HSC compartment and increase the chances of additional events that would promote leukemogenesis to occur.

Base editing and prime editing are the recent promising and rigorous refinements of genome editing technologies which could provide and improve a patient specific mutation correction for *GATA2* mutations or any other gene mutations that predispose to hematological malignancies when potentials and risks of these tools are tested sufficiently prior to the actual patient treatments. In addition to their potential for gene therapy discussed in this review, CRISPR base and prime editing technologies are also fantastic tools for basic research to introduce additional predicted leukemia driver mutations to HSCs in *GATA2* haploinsufficiency models in order to identify their potential role in malignant transformation.

## Author Contributions

CK and EP wrote the manuscript.

## Conflict of Interest

The authors declare that the research was conducted in the absence of any commercial or financial relationships that could be construed as a potential conflict of interest.
